# Histone deacetylase inhibitors regulate P-gp expression in colorectal cancer via transcriptional activation and mRNA stabilization

**DOI:** 10.18632/oncotarget.10488

**Published:** 2016-07-08

**Authors:** Hao Wang, Cheng Huang, Liang Zhao, Huan Zhang, Jing Mo Yang, Peng Luo, Bing-Xiang Zhan, Qing Pan, Jun Li, Bao-Long Wang

**Affiliations:** ^1^ Department of Clinical Laboratory, Affiliated Provincial Hospital of Anhui Medical University, Hefei, China; ^2^ School of Pharmacy, Anhui Medical University, Hefei, China; ^3^ Department of Biochemistry and Molecular Biology, Sichuan Cancer Hospital and Institute, Chengdu, China; ^4^ Department of Pharmacy, Anhui Provincial Cancer Hospital (West Branch of Anhui Provincial Hospital), Hefei, China

**Keywords:** histone deacetylase inhibitors, P-gp, STAT3, stability, multidrug resistance

## Abstract

Histone deacetylase inhibitors (HDACIs) are emerging as a novel class of anti-tumor drugs. But the effect of HDACIs in tumors treatment has been disappointing, which mainly due to the acquisition of resistance to HDACIs. However, the underlying mechanisms have not been clearly understood. In this study, it was found that HDACIs SAHA and TSA increased P-gp expression in CRC cells, which has been well known to contribute to drug resistant. The mechanisms underlying these effects were investigated. We showed that HDACIs enhanced transcriptional activity of P-gp protein encoding gene ABCB1. HDACIs treatment also increased the protein and mRNA expression of STAT3, but not PXR, CAR, Foxo3a or β-catenin, which are known to be involved in ABCB transcription regulation. Interestingly, knockdown of STAT3 significantly attenuated HDACIs-induced P-gp up-regulation in colorectal cancer cells, suggesting that STAT3 plays a crucial role in HDACIs-up-regulated P-gp. Furthermore, this study revealed for the first time that HDACIs enhanced the stability of ABCB1 at post-transcriptional level. Taken together, these results proved that HDACIs induced P-gp expression by two distinct ways, transcriptional activation and mRNA stabilization. Our results suggested that more attention should be paid to the cancer treatment using HDACIs since they will induce multidrug resistance in cancer cells.

## INTRODUCTION

Colorectal cancer (CRC) is one of the three leading causes of mortality induced by malignancies in humans worldwide. Recently, research revealed that the incidence of CRC in worldwide has obviously increased [1]. The optimal treatment for CRC is surgical resection, while chemotherapy servers as a crucial adjuvant therapies for CRC treatment. Currently, the emergence of multidrug resistance (MDR), by which cancer cells are resistant to broad spectrum anticancer drugs, is a major impediment to the success of CRC chemotherapy [2]. It has been demonstrated that the development of MDR phenotype in cancer cells is often attributable to high expression of membrane transport proteins, especially P-glycoprotein (P-gp) [3].

P-gp, a transmembrane glycoprotein encoded by the ABCB1 (also called multiple drug resistance 1, MDR1) gene, is the most important member of the ATP-binding cassette (ABC) transporters family. P-gp is an ATP-dependent efflux pump which decreases the intracellular concentration of a variety of anticancer drugs and is involved in the multidrug resistance [4]. Increasing evidences indicated that the activity and expression of efflux ABC transporters can be rapidly induced after exposure to some xenobiotics [5, 6]. The expression of ABC transporters can be regulated both at transcriptional or post-transcriptional levels. At transcriptional level, two nuclear receptors mainly expressed in the liver, the pregnane X receptor (PXR) and the constitutive androstane receptor (CAR), are widely considered to play a vital role in the regulation of ABCB1 expression [7–9]. Furthermore, other specific ligand-activated transcription factors, such as STAT3, Foxo3a and β-catenin, have been proved that could up-regulate mRNA expression of ABCB1 by directly combined to its promoter [10–12]. Besides transcriptional regulation, ABCB1 has also been demonstrated can be controlled via post-transcriptional levels, such as mRNA stability alteration [13, 14].

Histone deacetylase inhibitors (HDACIs) are appreciated as one of novel class of anti-tumor drugs tested clinically to cure a variety of cancers, including colorectal cancer. Their anti-tumor activities include promoting cell cycle arrest, differentiation and apoptosis, and inhibiting angiogenesis [15]. Several HDACIs, such as Trichostatin A (TSA) and vorinostat (suberolanilide hydroxamic acid, SAHA) have shown potent anti-tumor activities [16]. Clinically, SAHA has been approved by USFDA for treatment of cutaneous T-cell lymphoma [17]. As a potent anti-tumor drug, HDACIs have broad-spectrum antitumor activity and hypotoxicity in normal cells [18]. Moreover, HDACIs have been demonstrated to exhibit synergy with a variety of antitumor drugs, including cisplatin, doxorubicin, etoposide, paclitaxel and gemcitabine [19].

Despite HDACIs serve as an emerging class of potent anti-tumor drug, a few researches have proved that treatment cancer cells with HDACIs can give rise to broadspectrum anti-tumor MDR, leading to cells that are resistant to many functionally and structurally irrelevant drugs [20]. However, the underlying mechanisms are not entirely clear. In this study, we showed that HDACIs SAHA and TSA increased P-gp expression in HCT116 and SW480 cells. We also demonstrated that HDACIs enhanced P-gp expression by two distinct ways, transcriptional activation and mRNA stabilization.

## RESULTS

### Effect of HDACIs on P-gp expression

**Figure 1:**
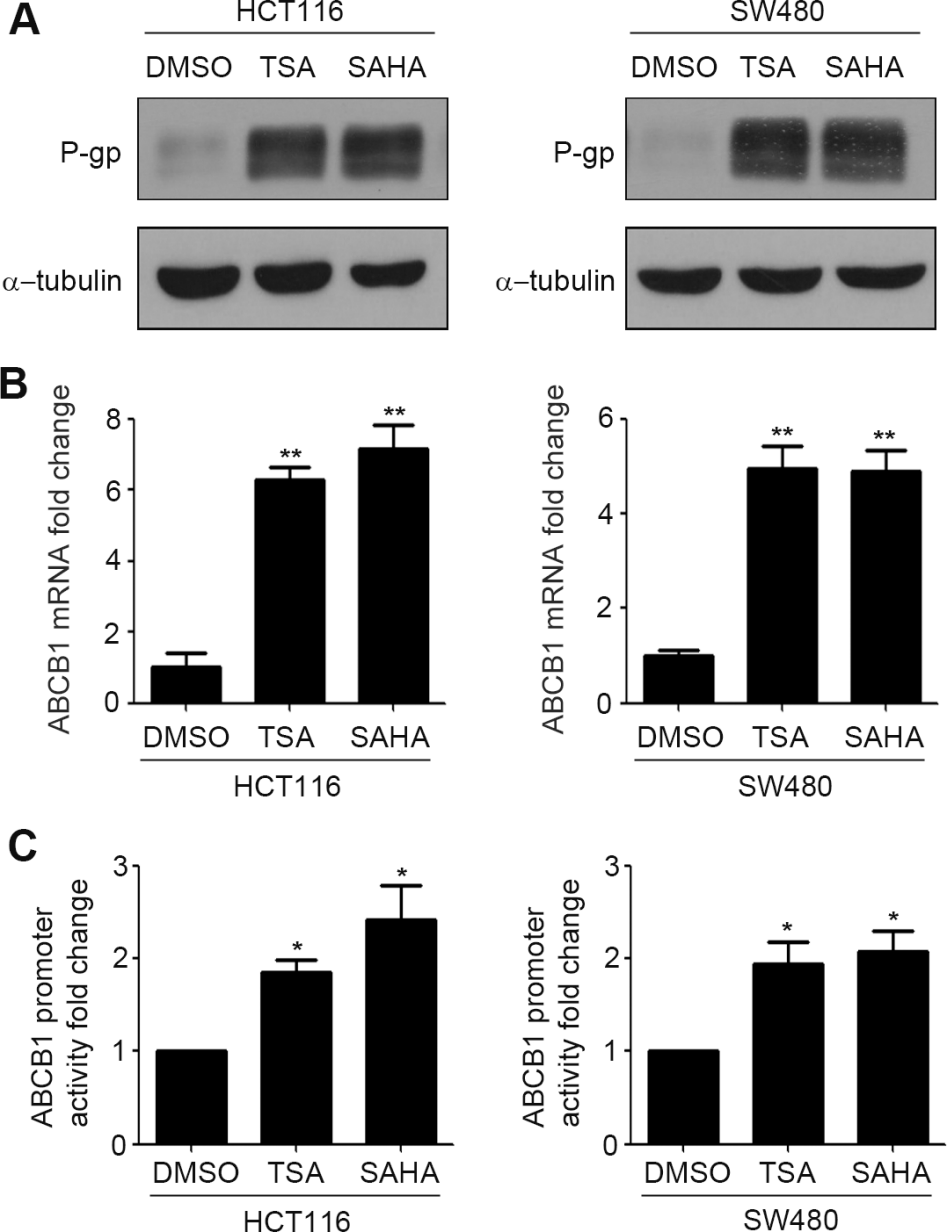
HDACIs induce P-gp expression in CRC cells (**A**–**B**) HCT116 and SW480 cells were treated with DMSO, SAHA (0.6 μM) or TSA (100 nM) for 24 h respectively, the expressions of P-gp protein was detected by western blot (A) and ABCB1 mRNA expression was detected by Quantitative RT-PCR (B),**p < 0.01. (**C**) The transcriptional activity of ABCB1 in HCT116 and SW480 cells treated with DMSO, SAHA (0.6 μM) or TSA (100 nM) were measured by dual luciferase report gene assay. *p < 0.05.

P-gp induces the ATP dependent efflux of a variety of chemotherapeutic agents [21]. To investigate the effect of HDACIs on P-gp expression, we analyzed the protein level of P-gp by western blotting in HCT116 and SW480 human colorectal carcinoma cell lines in the presence and absence of SAHA and TSA. As shown in Figure 1A, compared to DMSO, treatment with SAHA and TSA obviously increase the expression of P-gp protein. P-gp is encoded by ABCB1. We next detected mRNA level of ABCB1 in HCT116 and SW480 cells treated by SAHA and TSA. Results in Figure 1B showed that treatment with SAHA and TSA induce an increase in ABCB1 mRNA levels. Furthermore, we investigated whether HDACIs can influence transcriptional activity of ABCB1. The dual-luciferase reporter assay showed that SAHA and TSA obviously promote ABCB1 promoter transcriptional activity in HCT116 and SW480 cells compared with DMSO (Figure 1C).

### HDACIs induce drug resistance in CRC cells

**Figure 2:**
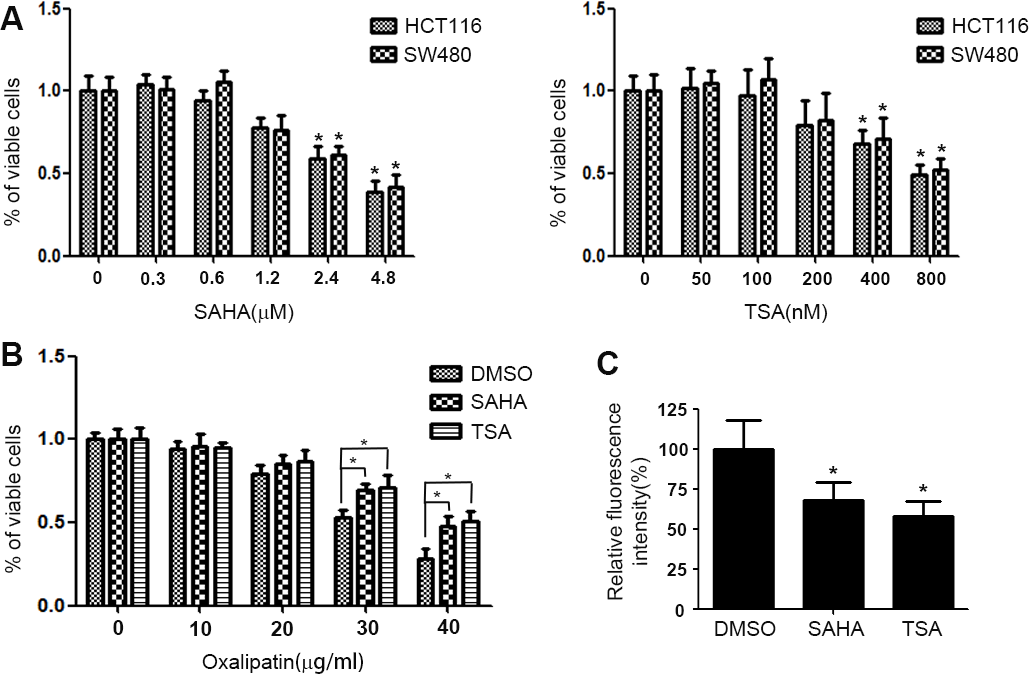
HDACIs induce drug resistance in CRC cells (**A**) HCT116 and SW480 cells were treated with various concentrations of SAHA or TSA for 48 h respectively. Cell viability was detected by MTT assay. *p < 0.05. (**B**) HCT116 cells were exposed to various concentrations of Oxalipatin for 24 h after 48 h treatment with DMSO, SAHA (0.6 μM) or TSA (100 nM) and cell viability was determined by MTT assay. *p < 0.05. (**C**) HCT116 cells were treated with DMSO, SAHA (0.6 μM) or TSA (100 nM) for 24 h. Following a change with fresh media containing 1 μM Rhodamine123, the cells were further incubated for 1 h. Then the cells were harvested and fluorescence intensity was determined by flow cytometry. *p < 0.05.

To discuss the effect of HDACIs on drug resistance, MTT assay was performed. HCT116 and SW480 cells were treated with various concentrations of SAHA or TSA for 48 h then the cell viability was determined by MTT assay. As shown in Figure 2A, 0.6 μM SAHA and 100 nM TSA almost have no effect on cell viability. Further, HCT116 cells were exposed to various concentrations of Oxalipatin for 24 h after 48 h treatment with DMSO, SAHA or TSA and cell viability was determined. We found that compared to DMSO, treatment with SAHA and TSA obviously increase drug resistance in HCT116 cells (Figure 2B). To investigate if the increase in P-gp expression was accompanied by induction of a functional P-gp, we determined the effect of SAHA and TSA on the intracellular accumulation of Rhodamine123, a fluorescent P-gp substrate. As shown in Figure 2C, SAHA and TSA attenuated the intracellular accumulation of Rhodamine123. These results indicated that induction of P-gp expression by SAHA and TSA can efficiently release drugs from the cells, which lead to the acquirement of drug resistance.

### HDACIs increase the expression of STAT3

**Figure 3:**
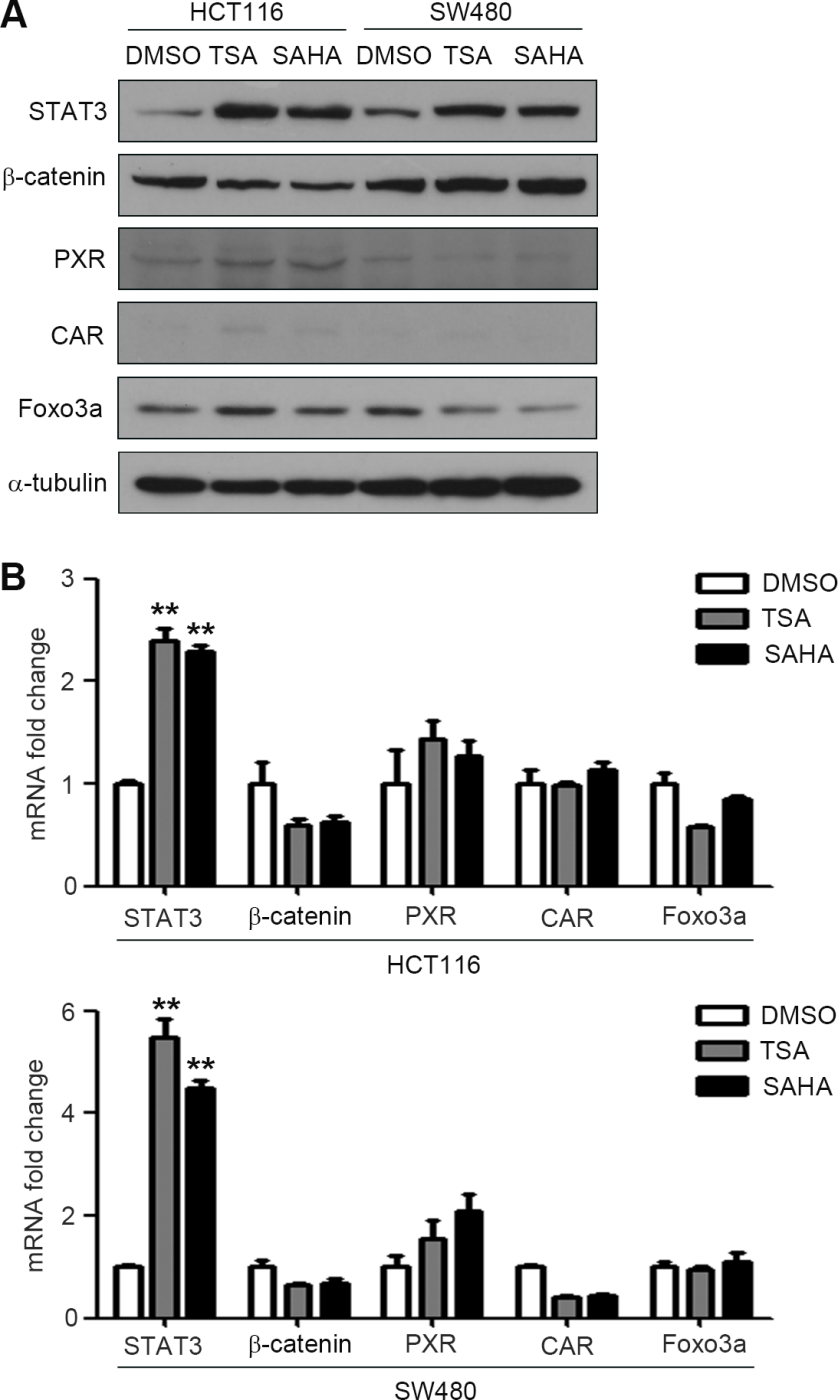
HDACIs induce STAT3 expression in CRC cells (**A**–**B**) HCT116 and SW480 cells were treated with DMSO, SAHA (0.6 μM) or TSA (100 nM) for 24 h respectively, the protein and mRNA expressions of STAT3, PXR, CAR, Foxo3a and β-catenin were detected by western blot (A) and Quantitative RT-PCR (B),**p < 0.01.

We further discussed the underlying mechanism of HDACIs-induced P-gp expression. As HDACIs can enhance transcriptional activity of ABCB1, we detected the expressions of a series of proteins which were reported can increase P-gp expression by directly binding to the promoter of ABCB1, such as STAT3, β-catenin, PXR, CAR, Foxo3a, etc. The expressions of these proteins were detected by western blotting in HCT116 and SW480 cells treatment with SAHA and TSA. Interestingly, the results showed that SAHA and TSA distinctly increase the expression of STAT3, but not the others (Figure 3A). We next measured the mRNA expressions of these proteins in HCT116 and SW480 cells treatment with SAHA and TSA. Similarly, SAHA and TSA increase the mRNA level of STAT3, but have no significant effect on the other proteins (Figure 3B). Based on these results, we assessed that STAT3 may be crucial for HDACIs-induced P-gp expression in HCT116 and SW480 cells.

### HDACIs increase the activity of STAT3

**Figure 4:**
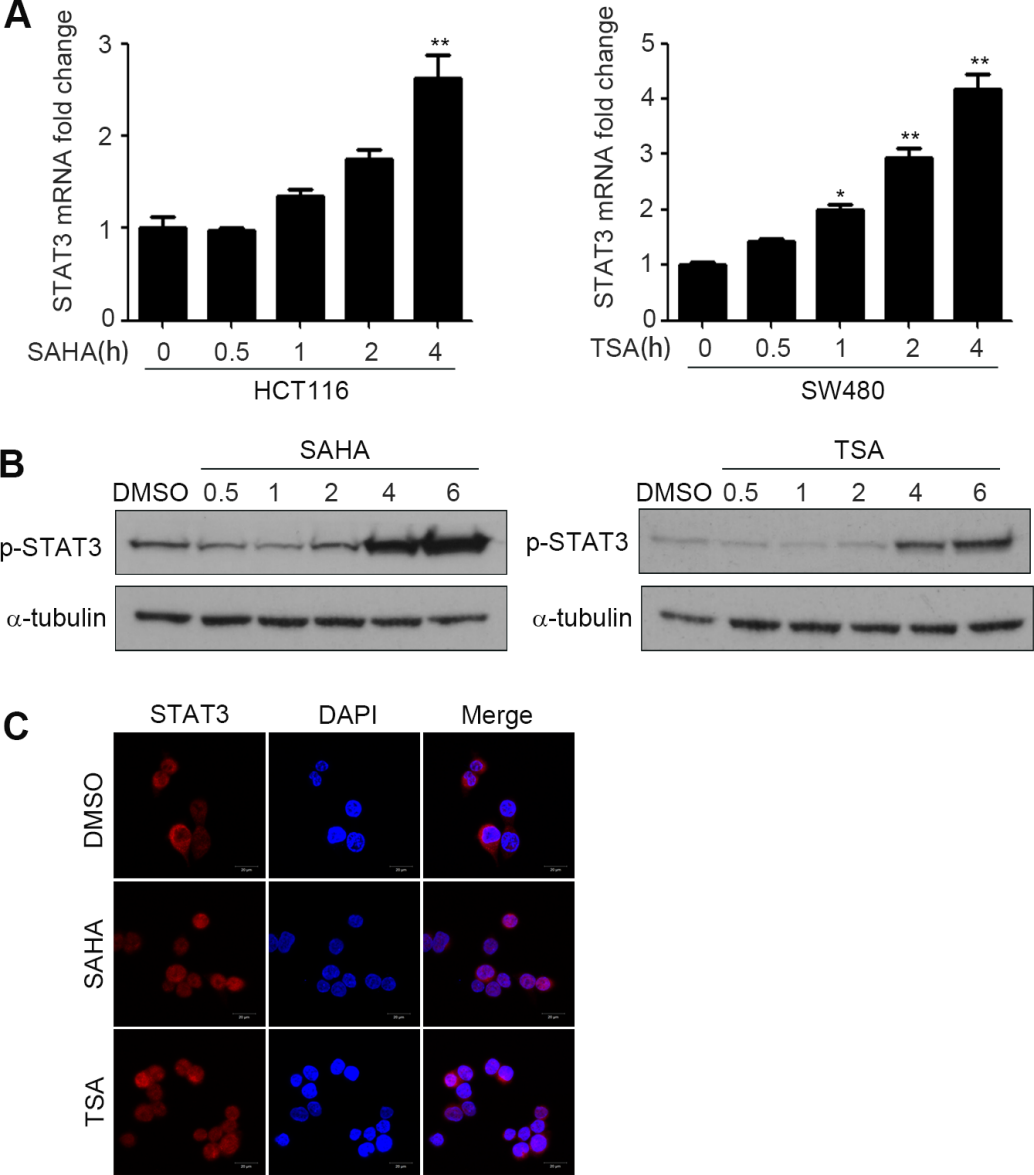
HDACIs enhance activity of STAT3 and promote its nuclear translocation (**A**) HCT116 and SW480 cells were treated with SAHA (0.6 μM) or TSA (100 nM) for 0–4 h, and STAT3 mRNA expression was detected by Quantitative RT-PCR, *p < 0.05, **p < 0.01. (**B**) HCT116 cells were treated with SAHA (0.6 μM) or TSA (100 nM) for 0.5–6 h, and the expression of p-STAT3 was detected by western blot. (**C**) HCT116 cells were treated with DMSO, SAHA (0.6 μM) or TSA (100 nM) for 6 h. After fixation, the cellular location of STAT3 (red) was examined by immunofluorescence staining and nuclei were stained with DAPI (blue). Scale bars: 20 μm.

To further verify the results in Figure 2B, we treated HCT116 and SW480 with SAHA or TSA respectively for 0–4 h, and detected mRNA level of STAT3 in various time. The results showed that the mRNA level of STAT3 was enhanced rapidly after SAHA or TSA stimulation and increased time-dependently (Figure 4A). Before STAT3 transfers into the nucleus to regulate its target gene, it must be phosphorylated [22]. To determine whether the activation of STAT3 is influenced by HDACIs, we treated HCT116 cells with SAHA or TSA for 0.5–6 h, and then the expression of p-STAT3 were determined by western blotting. We found that SAHA and TSA increased the expression of p-STAT3 in a time-dependent manner (Figure 4B). As a transcription factor, STAT3 can only take effect when it transfers into the cell nucleus, we also determined the nuclear translocation of STAT3 by immunofluorescence. As shown in Figure 4C, compared with DMSO, SAHA and TSA significantly increased the nuclear translocation of STAT3.

### STAT3 is crucial for HDACIs-mediated P-gp expression

**Figure 5:**
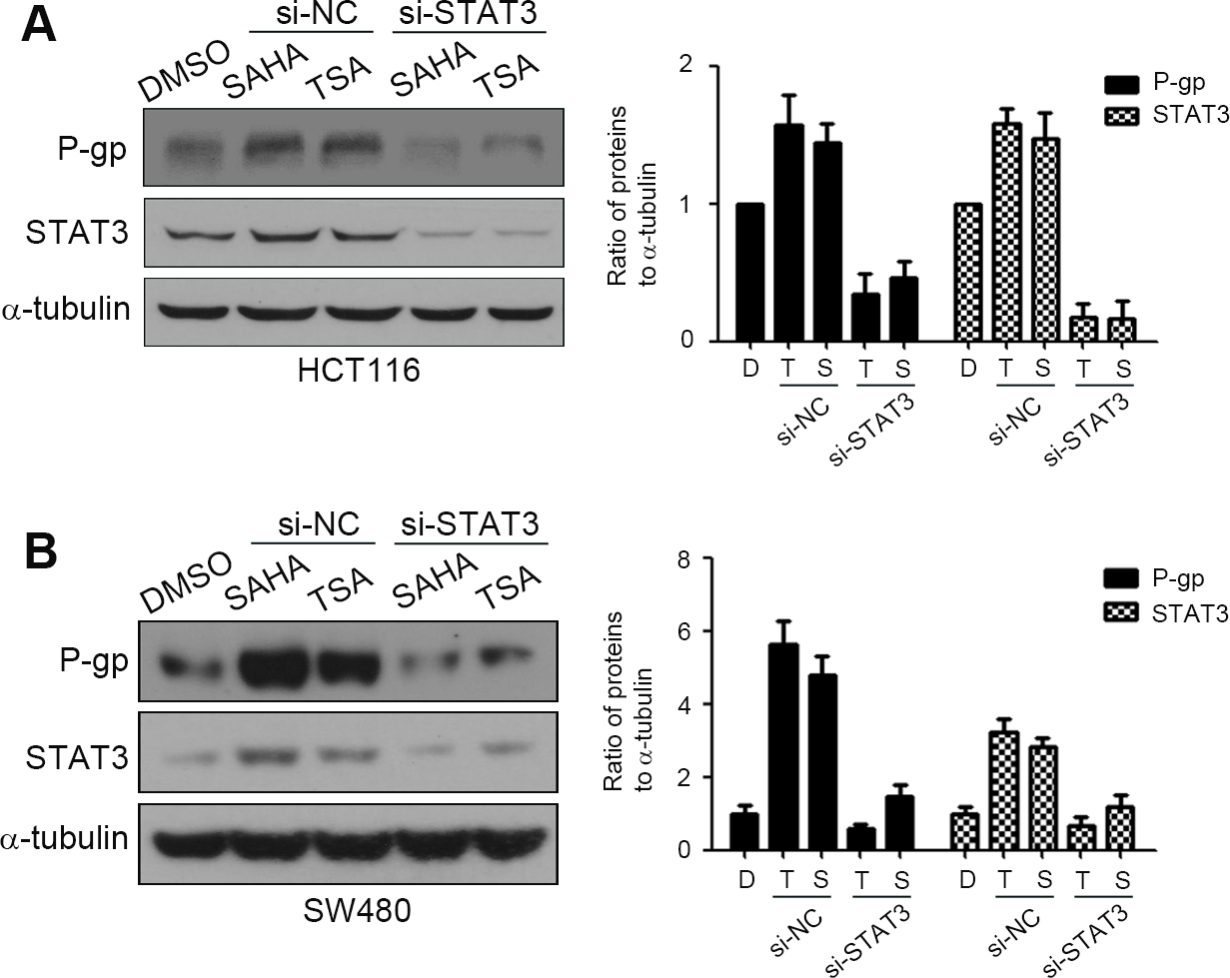
STAT3 is crucial for HDACIs-mediated P-gp up-regulation si-NC, negative control siRNA; si-STAT3, STAT3 siRNA. (**A**–**B**) HCT116 and SW480 cells were transfected with si-NC or si-STAT3 for 48 h, then treated with DMSO, SAHA (0.6 μM) or TSA (100 nM) for 24 h, the expression of P-gp and STAT3 were examined by western blotting.

To further verify the role of STAT3 in HDACIs-induced P-gp expression in colorectal cancer cells, the siRNAs were used to suppress the expression of STAT3. HCT116 and SW480 cells transfected with siRNAs were treated with SAHA or TSA and the expressions of P-gp and STAT3 were detected by western blotting. The results showed that silencing of STAT3 attenuated HDACIs-induced up-regulation of P-gp, which was not observed in control si-RNA-transfected cells (Figure 5A and 5B). Taken together, these observations demonstrated that STAT3 is essential for HDACIs-induced P-gp expression in colorectal cancer cells.

### HDACIs increase the mRNA stability of ABCB1

**Figure 6:**
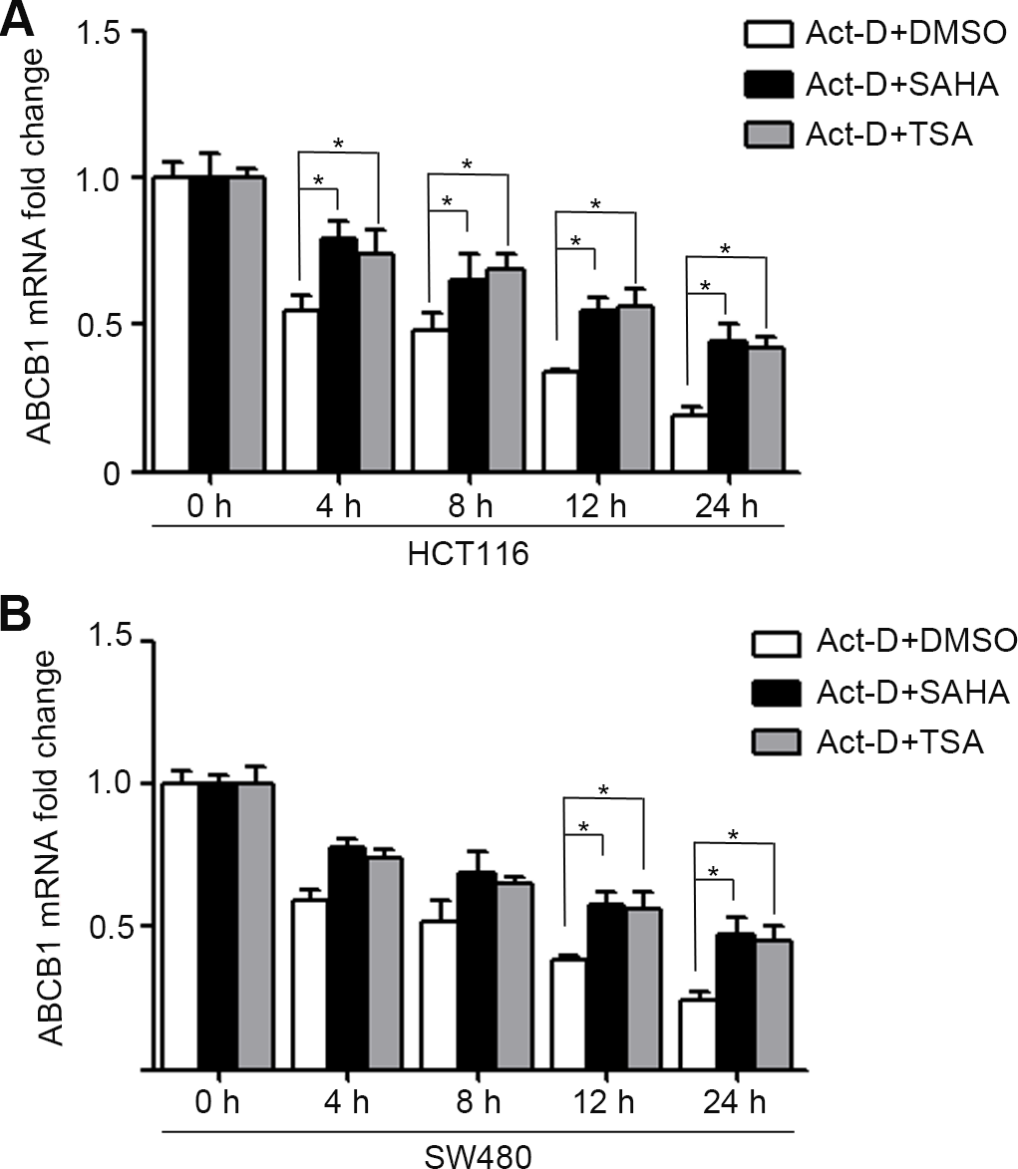
HDACIs enhance ABCB1 mRNA stability in CRC cells (**A**–**B**) HCT116 and SW480 cells were DMSO, SAHA (0.6 μM) or TSA (100 nM) for 24 h, after which the transcription inhibitor actinomycin D (Act-D 5 mg/ml for 1 h and 1 mg/ml for the following 24 h) was added to inhibit nascent RNA synthesis. Cells were harvested at various time-points (0, 4, 8, 12 or 24 h post Act-D treatment), total RNA was isolated ABCB1 mRNA level were determined by Quantitative RT-PCR, *p < 0.05.

A recent study found that P-gp expression in leukemic cells is regulated at two distinct steps, translational initiation and mRNA stabilization [23]. We have demonstrated that HDACIs can increase transcriptional activation of ABCB1 (Figure 1C). To further investigated whether HDACIs can affect the mRNA stability of ABCB1 in colorectal cancer cells, HCT116 and SW480 cells were treated with DMSO, SAHA or TSA for 24 h, after which the transcription inhibitor actinomycin D (Act-D) was added for the following 24 h to inhibit nascent RNA synthesis. Then the ABCB1 mRNA expression was detected by RT-PCR. The results showed that SAHA and TSA significantly enhanced the stability of ABCB1 in HCT116 and SW480 cells compare with DMSO (Figure 6A and 6B).

## DISCUSSION

The development of chemoresistance is a main impediment for any cancer therapy [24]. Despite HDACIs are known as a novel class of potent anti-cancer agent, several studies have found that HDACIs treatment can result in cancer cells resistant to many structurally and functionally irrelevant drugs, leading to cells acquire a broadspectrum anti-cancer MDR [20]. However, the underlying mechanisms are not clearly understood. In the present study, we showed that HDACIs SAHA and TSA increased P-gp expression in CRC cells, which has been well known to contribute to drug resistant in various cell lines. Previous studies have showed the up-regulation of Pgp in cancer cells exposed to HDACIs, including TSA and VPA [25, 26]. But the molecular mechanisms involved in HDACIs induced up-regulation of P-gp expression are not perfectly elucidated. Our study first reported that HDACIs SAHA and TSA induced P-gp expression by two distinct ways, transcriptional activation and mRNA stabilization.

Pgp is a membranebound transporter which can pump out drug metabolites and natural toxins, as well as anticancer drugs from the plasma membrane, which in turn gives rise to drug resistance [27]. We found that SAHA and TSA increased P-gp protein expression and its encoding gene ABCB1 mRNA expression (Figure 1A and 1B). Since transcriptional activation is the most common mechanism involved in the promotion of ABCB1 gene expression, we measured transcriptional activity of ABCB1 influenced by HDACIs. The results of dual luciferase reporter gene assay showed that SAHA and TSA could obviously enhance transcriptional activity of ABCB1 (Figure 1C). Several transcription factors have been characterized and identified in the regulation of ABCB1 promoter region. The nuclear receptors PXR and CAR have been proved that play an important role in ABCB1 gene tanscription regulation [28, 29] and are cellular biosensors capable of responding to chemical stimulation [30, 31]. Moreover, transcription factors β-catenin and Foxo3a have been reported that could increase ABCB1 mRNA expression by directly combined to its promoter [11, 12]. We investigated their potential role in the HDACIs-mediated up-regulation of ABCB1 gene. However, the results of western blotting and RT-PCR showed that SAHA and TSA treatment altered neither protein nor mRNA levels of PXR, CAR, β-catenin and Foxo3a (Figure 2A and 2B), suggesting that exist other mechanisms involved in up-regulation of ABCB1 transcription. A recent study demonstrated that transcription factor STAT3 was involved in the regulation of ABCB1 transcription by binding with its promoter region in leukemia cells [10]. Importantly, in our study, we found that SAHA and TSA treatment not only markedly increase STAT3 protein expression but also rapid up-regulate STAT3 mRNA expression in CRC cells (Figure 2A and Figure 4A). Furthermore, SAHA and TSA treatment enhanced STAT3 activity in a time-dependent manner and promoted its nuclear translocation (Figure 3A and 3B). To investigate the potential role of STAT3 in the HDACIs-mediated up-regulation of P-gp, STAT3 specific siRNA was used to knockdown the expression of STAT3. The results revealed that knockdown STAT3 inhibited HDACIs-induced up-regulation of P-gp in CRC cells (Figure 4A and 4B). Taken together, these observations proved that STAT3 played an important role in HDACIs-mediated up-regulation of P-gp in CRC cells. Therefore, STAT3 can be designed as a target for overcoming MDR of cancer cells induced by HDACIs in clinical cancer therapy.

Besides transcriptional activation, post-transcriptional regulation, such as mRNA stability alternation, was demonstrated to be an important process in P-gp expression [32]. Previous studies showed that a few of drugs, including doxorubicin, vinblastine, etoposide, colchicine and daunorubicin, can promote ABCB1 gene expression in mammalian cells by enhancing its mRNA stability [23, 33]. In addition, a recent research found that TSA could modulate Claudin-1 mRNA stability in CRC cells [34]. Therefore, we speculated that HDACIs may regulate ABCB1 mRNA stability in CRC cells. Interestingly, in our study, the stability of ABCB1 mRNA was indeed significantly enhanced by SAHA and TSA treatment, suggesting that HDACIs inhibited the degradation rate of ABCB1 mRNA thus contributing, at least in part, to the up-regulation of P-gp expression. This is, to our knowledge, the first study showing that HDACIs is able to increase ABCB1 mRNA stability. The particular mechanisms of ABCB1 mRNA stabilization in all of the above researches are unknown. The mRNA stability is tightly regulated by the interaction beteewn specific mRNA sequences and trans-acting factors, such as mRNA binding proteins and some microRNAs [6, 34]. Recently, mRNA binding proteins HuantigenR and Tristetraprolin have been reported play a crucial role in TSA-induced Claudin-1 mRNA stability [34]. Moreover, microRNAs have been implicated in the regulation of ABCB1 mRNA stability [35, 36]. Therefore, it is reasonable to speculate that HDACIs may activate a specific trans-acting factor, which then leading to increased ABCB1 mRNA stability. It is worth to further elucidate the detailed mechanisms of HDACIs-mediated ABCB1 mRNA stabilization.

In conclusion, this report found that as a novel class of anti-tumor drug, HDACIs is able to promote multi-drug resistance protein P-gp expression in CRC cells. Moreover, we investigated the underlying mechanisms of HDACIs-induced P-gp up-regulation, which is mainly via two distinct ways. On one hand, HDACIs up-regulate STAT3 expression, which then promote transcriptional activation of ABCB1. On the other hand, HDACIs enhance mRNA stability of ABCB1. Our results have significant clinical implications about drug disposition alteration and provide insight into the potential for development of multidrug resistance during CRC clinical treatment using HDACIs.

## MATERIALS AND METHODS

### Chemicals and reagents

TSA, SAHA and actinomycinD (Act-D) were obtained from Sigma-Aldrich (St Louis, MO). Primary antibodies against p-STAT3, STAT3, Foxo3a were obtained from Cell Signaling Technology (MA, USA). Primary antibodies against P-gp, PXR, CAR, β-catenin, α-tubulin were obtained from Santa Cruz Biotechnology (Santa Cruz, CA, USA). Horseradish peroxidase (HRP)-conjugated secondary antibody, Alexa Fluor 594 conjugated secondary antibody, DAPI and lipofectamine 2000 were purchased from Invitrogen (Carlsbad, CA, USA). PrimeScript^®^ RT reagent Kit and SYBR^®^ Premix Ex TaqTM were products of TaKaRa. E.Z.N.A^®^ HP Total RNA Kit was bought from Omega Bio-Tek (Doraville, USA). Smartpool siRNA against human STAT3 was obtained from RIBOBIO.

### Cell culture

The HCT116 and SW480 colorectal carcinoma cell lines were obtained from the Type Culture Collection of the Chinese Academy of Sciences (Shanghai, China). HCT116 cells were maintained in McCoy'5a culture medium (Gibco BRL) supplemented with 10% fetal bovine serum, and SW480 cells were cultured in DMEM culture medium (Gibco BRL) supplemented with 10% fetal bovine serum under a humidified 5% CO_2_ atmosphere at 37°C in incubator.

### RNA interference

The cells were seeded on a 6-well plate (2 × 10^5^ cells/well) and left in culture until the next day. They were then transfected with 100 pmol siRNA oligomer mixed with lipofectamine 2000 reagent in serum reduced medium according to the manufacturer's instructions. Medium was changed to complete culture medium 6 h later, and the cells were incubated at 37°C in a CO_2_ incubator for another 24 h before harvest.

### Quantitative Real-Time PCR

Total mRNA of cells was extracted after treatment for the indicated time. First strand cDNA synthesis was generated from 500 ng of total RNA. Quantification of target and reference (GAPDH) genes was performed in triplicate on LightCycler^®^ 480 II (Roche, Applied Science). The primers used in each reaction were as follows: ABCB1 forward 5′-TGCTCAGACAGGATGTGAGTTG-3′ andreverse 5′-AATTACAGCAAGCCTGGAACC-3′;STAT3 forward 5′-ACATTCTGGGCACAAACACA-3′ and reverse 5′-CAGTCACAATCAGGGAAGCA-3′; β-catenin forward 5′-GATAACCTGGATGCCGTCGTG-3′ and reverse 5′-CTTCACGCTCTTGAGACTTTGGTTC-3′; PXR forward 5′-CAAGCGGAAGAAAAGTGAAC-3′ and reverse 5′-TGAAATGGGAGAAGGTAGTG-3′; CAR forward 5′-ACTTTCTGTCTCCAAACACA-3′ and reverse 5′-GCAACTCCAAAAACTCTACC-3′; Foxo3a forward 5′-GCGTGCCCTACTTCAAGGA-3′ and reverse 5′-GACCCGCATGAATCGACTATG-3′; GAPDH forward 5′- GCACCGTCAAGGCTGAGAAC-3′ and reverse 5′-TGGTGAAGACGCCAGTGGA-3′. Following normalization to GAPDH gene, expression levels for target gene were calculated using the comparative threshold cycle (CT) method. The Δct values were calculated according to the formula Δct = ct (gene of interest)-ct (GAPDH) in correlation analysis, and the 2-ΔΔct was calculated according to the formula ΔΔct = Δct (control group)-Δct (experimental group) for determination of relative. Data is presented as the mean ± standard deviation (SD) from three independent experiments.

### Western blotting analysis

The cells were washed three times with ice-cold phosphate buffer solution (PBS) and then lysed in lysis buffer containing 50 mM Tris-HCl (pH 7.6), 150 mM NaCl, 1 mM EDTA, 1% NP-40, 0.5% Na-deoxycholate, 5 μg/ml aprotinin, 5 μg/ml leupeptin, and 1mM phenylmethylsulfonyl fluoride. Lysates were cleared by centrifugation and denatured by boiling in Laemmli buffer. Equal amounts of protein samples were loaded per well and separated on SDS-polyacrylamide gels, and then electrophoretically transferred onto PVDF membranes. Following blocking with 5% non-fat milk at room temperature for 2 h, membranes were incubated with primary antibodies (1:1,000 dilution) at 4°C overnight and then incubated with HRP-conjugated secondary antibodies (1:5,000 dilution) for 2 h at room temperature. Specific immune complexes were detected using Western Blotting Plus Chemiluminescence Reagent (Life Science).

### Immunofluorescence

The cells were cultured on chamber slides for 24 h, then washed three times with PBS, fixed with 4% paraformaldehyde for 20 min and permeabilized with 0.3% Triton X-100 for 10 min. After blocking with goat serum for 2 h at room temperature, cells were incubated with antibodies against STAT3 (1:100 dilution) at 4°C overnight. Slides were washed three times with PBS and incubated with Alexa Fluor 594-conjugated secondary antibodies (1:1,000 dilution) for 1 h at room temperature. Nuclei were stained with DAPI (10 μg/ml) for 10 min. Samples were examined with Confocal Laser Scanning Microscopy (Zeiss) to analyze location of STAT3.

### ABCB1 reporter assay

Cells were transiently co-transfected with pABCB1-luc (2 μg) and pRL-TK (0.5 μg). After 24 h, these cells were treated with TSA or SAHA for the indicated time. Transcriptional activity was determined by the dual-luciferase reporter assay system. Results were calculated as the ratio between the activity of pABCB1-luc and pRL-TK.

### MTT assay

Cell viability was performed using the MTT assay. Briefly, cells (7.5 × 10^3^) were seeded into each well of a 96-well plate and treated with Oxaliplation SAHA or TSA. After treatment, the cells were washed twice with PBS, and 100 ml of 0.25 mg/ml MTT inculture medium was added to each well. The plate was incubated at 37°C for 4 h. Then, the culture medium was removed, and 100 ml DMSO was added to each well to dissolve the dark blue crystal. The absorbance was measured at 570 nm using a microplate reader.

### Rhodamine123 accumulation assay

Fluorescence intensity of intracellular Rhodamine123 was measured by flow cytometry. Briefly, cells were treated with 1 μM Rhodamine123 for 1 h and harvested. Then the cells were washed with PBS for twice and the mean fluorescence intensity of intracellular Rhodamine123 was determined using flow cytometry.

### Statistical analysis

Results are expressed as Mean ± SD of three independent experiments unless otherwise specified. Data were analyzed by two-tailed unpaired Student's *t*-test between any two groups. One-way ANOVA analysis of variance was used to assess the difference of means among groups. These analyses were performed using GraphPad Prism Software Version 5.0 (GraphPad Software Inc., La Jolla, CA). A *P*-value of < 0.05 was considered statistically significant.

## References

[1] Saika K, Sobue T (2013). Cancer statistics in the world. Gan to kagaku ryoho. Cancer & chemotherapy.

[2] Ambudkar SV, Dey S, Hrycyna CA, Ramachandra M, Pastan I, Gottesman MM (1999). Biochemical, cellular, and pharmacological aspects of the multidrug transporter. Annu Rev Pharmacol.

[3] Gottesman MM, Fojo T, Bates SE (2002). Multidrug resistance in cancer: role of ATP-dependent transporters. Nat Rev Cancer.

[4] Schinkel AH, Jonker JW (2012). Mammalian drug efflux transporters of the ATP binding cassette (ABC) family: an overview. Adv Drug Deliver Rev.

[5] Abolhoda A, Wilson AE, Ross H, Danenberg PV, Burt M, Scotto KW (1999). Rapid activation of MDR1 gene expression in human metastatic sarcoma after *in vivo* exposure to doxorubicin. Clin Cancer Rev.

[6] Menez C, Mselli-Lakhal L, Foucaud-Vignault M, Balaguer P, Alvinerie M, Lespine A (2012). Ivermectin induces P-glycoprotein expression and function through mRNA stabilization in murine hepatocyte cell line. Biochem Pharmacol.

[7] Maglich JM, Stoltz CM, Goodwin B, Hawkins-Brown D, Moore JT, Kliewer SA (2002). Nuclear pregnane x receptor and constitutive androstane receptor regulate overlapping but distinct sets of genes involved in xenobiotic detoxification. Mol Pharmacol.

[8] Synold TW, Dussault I, Forman BM (2001). The orphan nuclear receptor SXR coordinately regulates drug metabolism and efflux. Nat Med.

[9] Wei P, Zhang J, Dowhan DH, Han Y, Moore DD (2002). Specific and overlapping functions of the nuclear hormone receptors CAR and PXR in xenobiotic response. Pharmacogenomics J.

[10] Zhang X, Xiao W, Wang L, Tian Z, Zhang J (2011). Deactivation of Signal Transducer and Activator of Transcription 3 Reverses Chemotherapeutics Resistance of Leukemia Cells via Down-Regulating P-gp. PloS one.

[11] Zhang H, Zhang X, Wu X, Li W, Su P, Cheng H, Xiang L, Gao P, Zhou G (2012). Interference of Frizzled 1 (FZD1) reverses multidrug resistance in breast cancer cells through the Wnt/beta-catenin pathway. Cancer Lett.

[12] Hui RCY, Francis RE, Guest SK, Costa JR, Gomes AR, Myatt SS, Brosens JJ, Lam EWF (2008). Doxorubicin activates FOXO3a to induce the expression of multidrug resistance gene ABCB1 (MDR1) in K562 leukemic cells. Mol Cancer Ther.

[13] Rodrigues AC, Curi R, Hirata MH, Crespo Hirata RD (2009). Decreased ABCB1 mRNA expression induced by atorvastatin results from enhanced mRNA degradation in HepG2 cells. Eur J Pharm Sci.

[14] Lee W, Choi HI, Kim MJ, Park SY (2011). Depletion of mitochondrial DNA up-regulates the expression of MDR1 gene via an increase in mRNA stability. Exp Mol Med.

[15] Ni X, Li L, Pan G (2015). HDAC inhibitor-induced drug resistance involving ATP-binding cassette transporters. Oncol Lett.

[16] Chavan AV, Somani RR (2010). HDAC Inhibitors-New Generation of Target Specific Treatment. Mini-Rev Med Chem.

[17] Duvic M, Vu J (2007). Vorinostat: a new oral histone deacetylase inhibitor approved for cutaneous T-cell lymphoma. Expert Opin Inv Drug.

[18] Botrugno OA, Santoro F, Minucci S (2009). Histone deacetylase inhibitors as a new weapon in the arsenal of differentiation therapies of cancer. Cancer Lett.

[19] Stimson L, La Thangue NB (2009). Biomarkers for predicting clinical responses to HDAC inhibitors. Cancer Lett.

[20] Ambudkar SV, Kimchi-Sarfaty C, Sauna ZE, Gottesman MM (2003). P-glycoprotein: from genomics to mechanism. Oncogene.

[21] Caceres G, Robey RW, Sokol L, McGraw KL, Clark J, Lawrence NJ, Sebti SM, Wiese M, List AF (2012). HG-829 Is a Potent Noncompetitive Inhibitor of the ATP-Binding Cassette Multidrug Resistance Transporter ABCB1. Cancer Res.

[22] Subramaniam A, Shanmugam MK, Perumal E, Li F, Nachiyappan A, Dai XY, Swamy SN, Ahn KS, Kumar AP, Tan BKH, Hui KM, Sethi G (2013). Potential role of signal transducer and activator of transcription (STAT)3 signaling pathway in inflammation, survival, proliferation and invasion of hepatocellular carcinoma. BBA-Rev Cancer.

[23] Yague E, Armesilla AL, Harrison G, Elliott J, Sardini A, Higgins CF, Raguz S (2003). P-glycoprotein (MDR1) expression in leukemic cells is regulated at two distinct steps, mRNA stabilization and translational initiation. J Biol Chem.

[24] Wang H, Zhang G, Zhang H, Zhang F, Zhou BH, Ning F, Wang HS, Cai SH, Du J (2014). Acquisition of epithelial-mesenchymal transition phenotype and cancer stem cell-like properties in cisplatin-resistant lung cancer cells through AKT/beta-catenin/Snail signaling pathway. Eur J Pharmaco.

[25] Hauswald S, Duque-Afonso J, Wagner MM, Schertl FM, Luebbert M, Peschel C, Keller U, Licht T (2009). Histone Deacetylase Inhibitors Induce a Very Broad, Pleiotropic Anticancer Drug Resistance Phenotype in Acute Myeloid Leukemia Cells by Modulation of Multiple ABC Transporter Genes. Clin Cancer Res.

[26] Eyal S, Lamb JG, Smith-Yockman M, Yagen B, Fibach E, Altschuler Y, White HS, Bialer M (2006). The antiepileptic and anticancer agent, valproic acid, induces P-glycoprotein in human tumour cell lines and in rat liver. Brit J Pharmacol.

[27] Fukuda Y, Schuetz JD (2012). ABC transporters and their role in nucleoside and nucleotide drug resistance. Biochem Pharmacol.

[28] Burk O, Arnold KA, Geick A, Tegude H, Eichelbaum M (2005). A role for constitutive androstane receptor in the regulation of human intestinal MDR1 expression. J Biol Chem.

[29] Kota BP, Tran VH, Allen J, Bebawy M, Roufogalis BD (2010). Characterization of PXR mediated P-glycoprotein regulation in intestinal LS174T cells. Pharmacol Res.

[30] Timsit YE, Negishi M (2007). CAR and PXR: The xenobiotic-sensing receptors. Steroids.

[31] Tolson AH, Wang HB (2010). Regulation of drug-metabolizing enzymes by xenobiotic receptors: PXR and CAR. Adv Drug Deliver Rev.

[32] Lee CH, Bradley G, Ling V (1998). Increased P-glycoprotein messenger RNA stability in rat liver tumors *in vivo*. J Cell Physiol.

[33] Baker EK, Johnstone RW, Zalcberg JR (2005). El-Osta A, Epigenetic changes to the MDR1 locus in response to chemotherapeutic drugs. Oncogene.

[34] Sharma A, Bhat AA, Krishnan M, Singh AB, Dhawan P (2013). Trichostatin-A modulates claudin-1 mRNA stability through the modulation of Hu antigen R and tristetraprolin in colon cancer cells. Carcinogenesis.

[35] Kovalchuk O, Filkowski J, Meservy J, Iinytskyy Y, Tryndyak VP, Chekhun VF, Pogribny IP (2008). Involvement of microRNA-451 in resistance of the MCF-7 breast cancer cells to chemotherapeutic drug doxorubicin. Mol Cancer Ther.

[36] Zhu H, Wu H, Liu XP, Euans BR, Medina DJ, Liu CG, Yang JM (2008). Role of MicroRNA miR-27a and miR-451 in the regulation of MDR1/P-glycoprotein expression in human cancer cells. Biochem Pharmacol.

